# Two New Compounds Containing Pyridinone or Triazine Heterocycles Have Antifungal Properties against *Candida albicans*

**DOI:** 10.3390/antibiotics11010072

**Published:** 2022-01-08

**Authors:** Laura Mena, Muriel Billamboz, Rogatien Charlet, Bérangère Desprès, Boualem Sendid, Alina Ghinet, Samir Jawhara

**Affiliations:** 1 UMR 8576—UGSF—Unité de Glycobiologie Structurale et Fonctionnelle, Centre National de la Recherche Scientifique, Institut National de la Santé et de la Recherche Médicale U1285, University of Lille, F-59000 Lille, France; lauramena@outlook.fr (L.M.); charlet-rogatien@hotmail.fr (R.C.); dpsberengere@gmail.com (B.D.); boualem.sendid@univ-lille.fr (B.S.); 2 Medicine Faculty, University of Lille, F-59000 Lille, France; 3 CHU Lille, Service de Parasitologie Mycologie, Pôle de Biologie Pathologie Génétique, F-59000 Lille, France; 4 Institut National de la Santé et de la Recherche Médicale, CHU Lille, Institut Pasteur de Lille, U1167—RID-AGE—Facteurs de Risque et Déterminants Moléculaires des Maladies Liées au Vieillissement, University of Lille, F-59000 Lille, France; muriel.billamboz@junia.com (M.B.); alina.ghinet@junia.com (A.G.); 5 JUNIA, Health and Environment, Laboratory of Sustainable Chemistry and Health, F-59000 Lille, France; 6 Faculty of Chemistry, ‘Alexandru Ioan Cuza’ University of Iasi, Bd. Carol I, nr. 11, 700506 Iasi, Romania

**Keywords:** *Candida albicans*, antifungal agents, biofilm formation, *C. elegans* infection model

## Abstract

Candidiasis, caused by the opportunistic yeast *Candida albicans*, is the most common fungal infection today. Resistance of *C. albicans* to current antifungal drugs has emerged over the past decade leading to the need for novel antifungal agents. Our aim was to select new antifungal compounds by library-screening methods and to assess their antifungal effects against *C. albicans*. After screening 90 potential antifungal compounds from JUNIA, a chemical library, two compounds, 1-(4-chlorophenyl)-4-((4-chlorophenyl)amino)-3,6-dimethylpyridin-2(1*H*)-one (PYR) and (*Z*)-*N*-(2-(4,6-dimethoxy-1,3,5-triazin-2-yl)vinyl)-4-methoxyaniline (TRI), were identified as having potential antifungal activity. Treatment with PYR and TRI resulted in a significant reduction of *C. albicans* bioluminescence as well as the number of fungal colonies, indicating rapid fungicidal activity. These two compounds were also effective against clinically isolated fluconazole- or caspofungin-resistant *C. albicans* strains. PYR and TRI had an inhibitory effect on *Candida* biofilm formation and reduced the thickness of the mannan cell wall. In a *Caenorhabditis elegans* infection model, PYR and TRI decreased the mortality of nematodes infected with *C. albicans* and enhanced the expression of antimicrobial genes that promote *C. albicans* elimination. Overall, PYR and TRI showed antifungal properties against *C. albicans* by exerting fungicidal activities and enhancing the antimicrobial gene expression of *Caenorhabditis elegans*.

## 1. Introduction

Opportunistic fungi have become an increasingly important cause of nosocomial bloodstream infections, with high rates of morbidity and mortality in intensive care units [1,2]. These fungal infections are particularly problematic for immunocompromised patients, as well as patients with solid-organ malignancies or those recovering from abdominal surgery [1,2]. *Candida albicans* is an opportunistic yeast that colonizes the oropharyngeal, esophageal, and gastrointestinal mucosa in most healthy humans. Overgrowth of *C. albicans* in these niches can result in mucosal infections and life-threatening systemic disease, making *C. albicans* an important opportunistic pathogen. *C. albicans* is the most commonly identified *Candida* species in clinical settings and is one of the leading causes of hospital-acquired infections. Antifungal resistance is an emerging problem worldwide and this complicates the selection of appropriate antifungal therapy [3,4]. A subset of *Candida* strains are now resistant to first-line antifungal agents such as fluconazole and echinocandins [4]. This increasing prevalence of strains that are resistant to current antifungal drugs correlates with high azole and echinocandin use in clinical wards hosting patients at high risk of invasive candidiasis.

*C. albicans* is capable of adhering to catheters and other medical devices contributing to the formation of biofilms, which are heterogeneous structures containing hyphae, pseudohyphae, and yeast cells. These develop at the interface between an aqueous medium and a solid [5]. Biofilms are difficult for antifungals to penetrate and represent a potential source of candidemia relapses, leading to high rates of morbidity and mortality. Biofilms are therefore an important virulence factor and closely correlate with invasive fungal infections [6].

The nematode *Caenorhabditis elegans* has been widely used as a model for the study of *C. albicans* pathogenesis and the host immune system [7]. The *C. elegans* model enables the screening of different compounds for antifungal activity. The *C. elegans* immune response also plays a critical role during host–microbe interactions because many fungal and bacterial pathogens that cause illness in humans also cause tissue damage in *C. elegans* [8]. Antimicrobial peptides are the key components of innate immunity in *C. elegans*, including the carbohydrate-binding C-type lectin encoding clec gene repertoire, lysozymes, and caenacins [9,10]. In addition, a p38 mitogen activated kinase (MAPK) pathway, involving MAPK PMK-1, is among the different signaling pathways involved in modulating the innate immune response in *C. elegans* [10,11].

Recently, 2,3-dihydroxy-4-methoxybenzaldehyde (DHMB) was identified as a compound with potential antifungal activity against fluconazole- and caspofungin-resistant *C. albicans* [12]. DHMB also reduced the clinical and histological scores for inflammation in DSS-induced colitis in mice and promoted the elimination of *C. albicans* from the gut [12].

In the present study, we screened 90 small aromatic compounds from the JUNIA chemical library for their possible antifungal activity against *C. albicans*. Two compounds were identified as novel small molecules with antifungal activity against *Candida* viability, *Candida* biofilms, and cell wall fitness. The antifungal effects of these two compounds on *C. albicans* virulence and the host immune response were studied in a *C. elegans* infection model.

## 2. Results

### 2.1. Screening of the Chemical Library

**Figure 1:**
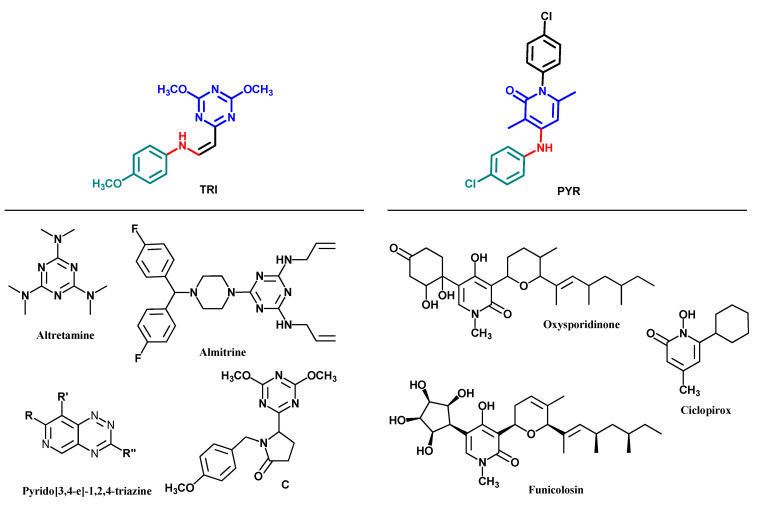
Active compounds TRI and PYR identified from initial screening; structure of compound C and reported active compounds similar to TRI and PYR.

Ninety compounds from the chemical library were screened for their antifungal and antibacterial activity. (*Z*)-*N*-(2-(4,6-dimethoxy-1,3,5-triazin-2-yl)vinyl)-4-methoxyaniline (TRI) and 1-(4-chlorophenyl)-4-((4-chlorophenyl)amino)-3,6-dimethylpyridin-2(1*H*)-one (PYR) were found to have antifungal activity against *C. albicans* (Figure 1). These two compounds shared some similar features, including a 6-membered *N*-heterocycle, a free -NH- link, and a *para*-substituted aromatic moiety (see Figure 1).

Some derivatives of TRI and PYR were also screened against *C. albicans* to determine the importance of each moiety in antifungal activity. In a first experiment, modification of the amine moiety was performed, while keeping the 4,6-dimethoxy-1,3,5-triazine and ethylene linkers constant (Table 1). Suppression of the *para*-methoxy substituent on the phenyl ring (TRI1) led to a decrease in activity, measured at 32 µg/mL and confirmed at 0.8 mmol/L (Table 1, entry 2). Polymethoxylated substitution or addition of a dioxolane ring did not improve the activity against *C. albicans*, whatever the position of the substituent on the aromatic ring (Table 1, entries 3–8). Introducing a 3-hydroxy-4-methoxyphenyl unit, similar to DHMB, an antifungal previously described by our group, did not result in any improvement in activity (Table 1, entry 6) [12]. Moreover, replacing the methoxy groups with chloro substituents did not increase the antifungal potency (Table 1, entry 9). Introducing diethylamine in compound TRI9 instead of *p*-anisidine of TRI slightly decreased the activity, which could be related to the importance of the aromatic ring or the H-bonding capacity of the -NH- link (Table 1, entry 10). To confirm the role of the -NH- link, compound C, in which the *p*-methoxyphenyl group was conserved but the amino-vinyl linker was modified, was also screened (Figure 1). Compound C was 6-fold less active than TRI against *C. albicans*. This highlights the importance of a -NH- linker for activity.

Derivatives of PYR compounds were also evaluated, with chemical modifications on the aromatic moieties (Table 1, series B). In this experiment, it is notable that the *para*-chloroaniline moiety had the highest activity against *C. albicans*. Introducing inductive or mesomeric electro-donating substituents such as methyl- or methoxy- on the aromatic rings led to a decrease in biological activity (Table 1, entries 12–16). Moreover, replacing one aromatic ring by a piperazine part as in compounds PYR7 and PYR8 did not improve the activity (Table 1, entries 17–18). While in series A, the *p*-methoxyaniline was the most effective ring for antifungal activity, it appeared that in series B, *p*-chloroaniline was the best choice. Gathering these data, compounds TRI and PYR were selected for further biological studies.

### 2.2. Effect of PYR and TRI on C. albicans Viability

**Figure 2:**
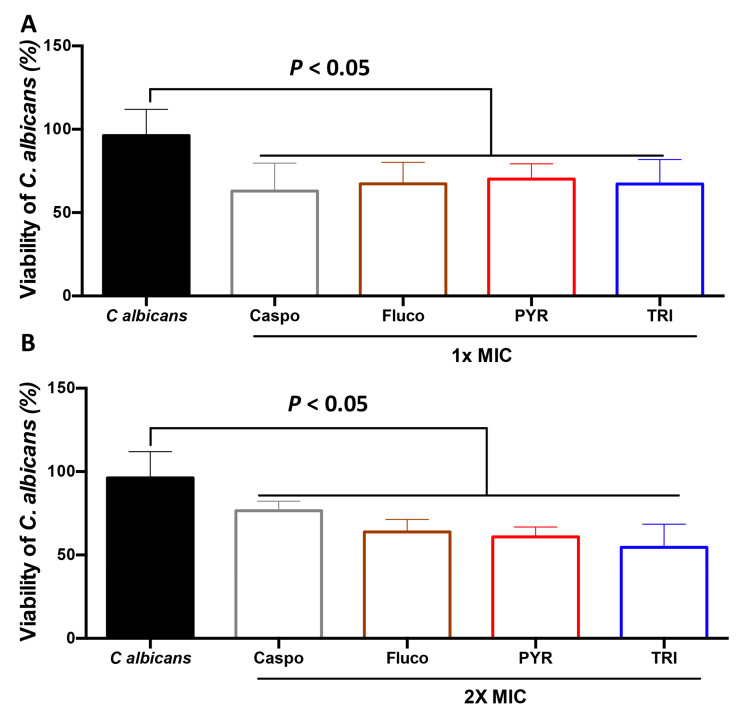
Viability of *C. albicans* by culture assay in the presence of caspofungin, fluconazole, PYR, or TRI. *C. albicans* SC5314 cells were challenged with caspofungin (Caspo), fluconazole (Fluco), PYR, or TRI at their (**A**) 1× MIC, or (**B**) 2× MIC. *C. albicans* (control) corresponds to *C. albicans* alone without any antifungal treatment. Error bars indicate standard deviations.

**Figure 3:**
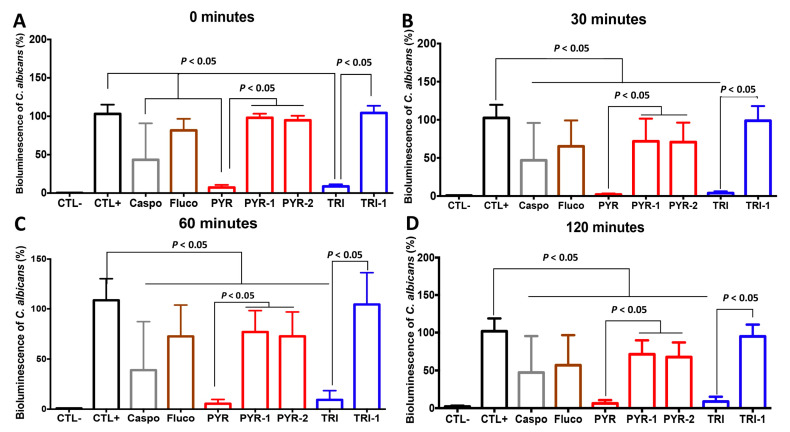
Impact of PYR and TRI and their derivatives on *C. albicans* viability. Bioluminescent *C. albicans* strain was treated with PYR, TRI, and their derivatives (PYR-1, PYR-2, and TRI-1) at their 1x MIC and monitored at (**A**) 0, (**B**) 30, (**C**) 60, and (**D**) 120 min. CTL corresponds to PBS and substrate. CTL+ represents *C. albicans* challenged with PBS. Caspo corresponds to *C. albicans* cells challenged with caspofungin (1× MIC). Fluco represents *C. albicans* cells challenged with fluconazole (1× MIC). Error bars indicate standard deviations.

To determine the antifungal effect of PYR and TRI on *C. albicans* viability, *C. albicans* cells were challenged with PYR or TRI and viability was assessed by culture-plate assay (Figure 2). PYR and TRI at their 1× MIC (12.5 µg/mL and 50 µg/mL, respectively) reduced *C. albicans* viability and this antifungal activity was more pronounced than with caspofungin at 2× MIC (Figure 2). Additionally, the viability of *C. albicans* cells was monitored in real time using a bioluminescent *C. albicans* strain challenged with either caspofungin, fluconazole, PYR, or TRI (at 0, 30, 60, and 120 min). PYR and TRI at their MICs (12.5 µg/mL and 50 µg/mL, respectively) led to a significant reduction in bioluminescence of *C. albicans* when compared to that of *C. albicans* unchallenged with antifungal compounds (Figure 3). This reduction was observed rapidly after *C. albicans* treatment with PYR and TRI indicating the fungicidal effect of these compounds against *C. albicans*. We then analyzed whether derivatives of PYR (PYR1 and PYR2) and TRI (TRI1) had antifungal activity against *C. albicans* (Figure 3). In contrast to TRI1, the derivatives PYR1 and PYR2 had antifungal activity against *C. albicans*. Of note, the derivatives PYR1 and PYR2 had less antifungal activity against *C. albicans* when compared to their original compound, PYR (Figure 3). These data confirmed that the substitution of the aromatic moiety is highly important in the activity. For PYR derivatives, *p*-chloro substituent is superior to the *p*-methoxy one whereas the *p*-methoxy group is needed for activity in the TRI series of compounds.

**Figure 4:**
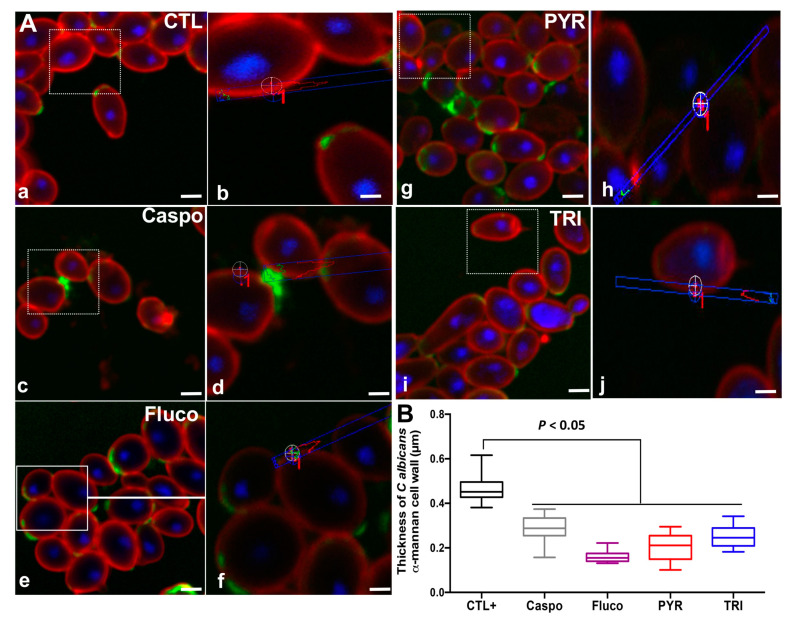
Modulation of chitin and α-mannan in the *C. albicans* cell wall after antifungal challenge. (**A**) Confocal microscopy of *C. albicans* SC5314 strain challenged with PBS (CTL: a and b), caspofungin (Caspo: c and d), fluconazole (Fluco: e and f), PYR (g and h), or TRI (i and j) at their 1× MIC for 2 h. α-mannans were labelled with concanavalin A-rhodamine (red) and chitin was labelled with WGA-IFTC (green). Nuclei were labelled with DAPI. Scale bars represent 5 µm for (a, c, e, g, and j) and 1 µm for (b, d, f, h, and j). Magnification 100× with 1.4 numerical aperture. (**B**) Thickness of *C. albicans* cell wall α-mannan (µm). Error bars indicate standard deviations.

To evaluate whether PYR and TRI had antifungal activity against drug-resistant *C. albicans* clinical isolates, we selected five *C. albicans* clinical isolates resistant to either fluconazole or caspofungin (Table 2 and Table 3). PYR and TRI significantly decreased the viability of drug-resistant *C. albicans* clinical isolates at 1× MIC and 2× MIC in terms of viable colony counts using fungal culture media (Table 3). We next assessed the effect of PYR and TRI on the *C. albicans* cell wall (Figure 4). Quantification of the fluorescence intensity of *C. albicans* cells labeled with concanavalin A from 30 different images extracted from confocal microscopy using ImageJ showed that PYR and TIR reduced the thickness of the mannan part of the *C. albicans* cell wall while the labeling of *C. albicans* with wheat germ agglutinin (WGA) did not show any changes in the chitin part of the cell wall (Figure 4). 

**Figure 5:**
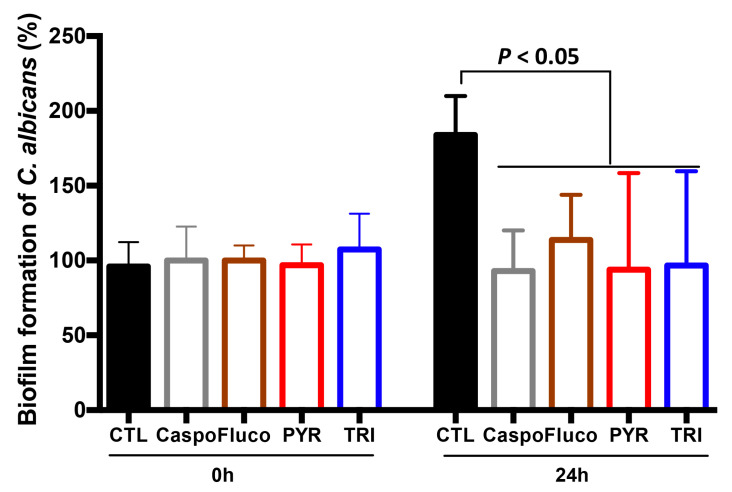
Effect of PYR and TRI on *C. albicans* biofilm formation. *C. albicans* SC5314 cells were allowed to form a biofilm for 24 h. *C. albicans* biofilms were treated with caspofungin (Caspo), fluconazole (Fluco), PYR, or TRI at their 1× MIC for 24 h. CTL represents *C. albicans* alone without any antifungal treatment. Error bars indicate standard deviations.

To determine whether PYR and TRI had an impact on biofilm formation, which is involved in resistance to antifungal agents by acting as a shield that delays or prevents drug diffusion to host cells, *C. albicans* biofilms were challenged with the two new antifungal compounds (Figure 5). The cell density of *C. albicans* challenged with caspofungin, fluconazole, PYR, or TRI at their MICs (0.03 µg/mL, 0.5 µg/mL, 12.5 µg/mL, and 50 µg/mL, respectively) was clearly lower when compared to that of untreated *C. albicans*. In this assay, the measurement of *C. albicans* cell-wall thickness was difficult from microscope images since the biofilm matrix was a dense and highly compact structure in all conditions (*C. albicans* alone or *C. albicans* challenged with antifungal compounds). 

### 2.3. PYR and TRI Reduced the Virulence of C. albicans in the C. elegans Infection Model

**Figure 6:**
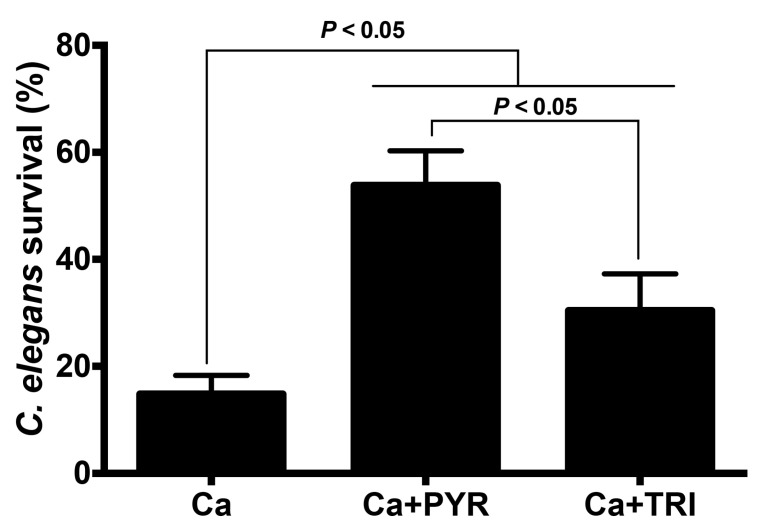
Effects of PYR and TRI on *C. albicans* virulence in *C. elegans*. Nematodes infected with *C. albicans* SC5314 strain were examined for survival daily for 4 days and the percentage nematode survival was determined on day 4. Nematodes were considered dead when they did not respond to being touched with a platinum-wire pick. Ca + PYR represents *C. elegans* infected with *C. albicans* and treated with PYR. Ca + TRI represents *C. elegans* infected with *C. albicans* and treated with TRI. Data are presented as mean ± SD of four independent measurements.

The effects of PYR and TRI on *C. albicans* virulence in vivo were investigated using a *C. elegans* nematode infection model. Nematodes infected with *C. albicans* were treated with either PYR or TRI (Figure 6). The survival of nematodes was followed daily by microscopic observation. *C. albicans* infection was observed to cause 85% mortality of *C. elegans* at day 4. PYR treatment of *C. elegans* infected with *C. albicans* at 12.5 µg/mL increased nematode survival to about 53% at day 4 while TRI treatment at 50 µg/mL protected the nematodes to about 30% survival indicating that PYR and TRI are active agents in prolonging nematode survival against *C. albicans* infection (Figure 6).

**Figure 7:**
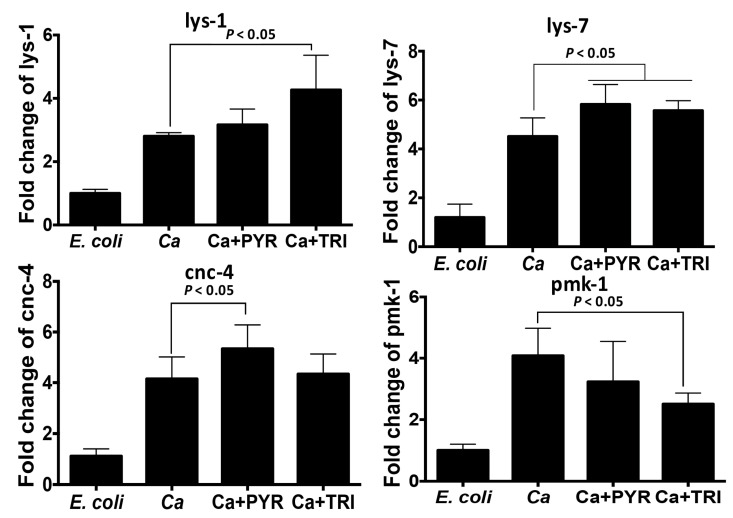
Relative expression of antimicrobial peptides (lys-1, lys-7, and cnc-4) and the immune gene (pmk-1) in *C. elegans* infected with *C. albicans*. L4 nematodes were infected with *C. albicans* SC5314 lawns for 6 h and then treated with PYR or TRI at their 1x MIC for 12 h. *E. coli* represents *C. elegans* fed with *E. coli*. Ca represents *C. elegans* infected with *C. albicans*. Ca + PYR corresponds to *C. elegans* infected with *C. albicans* and treated with PYR. Ca + TRI represents *C. elegans* infected with *C. albicans* and treated with PYR. Data are presented as mean ± SD of four independent measurements.

To explore the effect of these two compounds on the immune response of *C. elegans*, we studied the expression of lys-1, lys-7, and cnc-4 involved in the antimicrobial response and pmk-1 (p38 MAPK signaling pathway), which concerns the immune reaction. In comparison with *Escherichia coli* OP50 control conditions, *C. albicans* increased the expression of lys-1, lys-7, cnc-4, and pmk-1 (Figure 7). PYR treatment of *C. elegans* infected with *C. albicans* enhanced the expression of lys-7 and cnc-4 while no significant change in pmk-1 expression was observed. TRI treatment of *C. elegans* infected with *C. albicans* induced the up-regulation of lys-1, lys-7, and cnc-4 while a reduction of pmk-1 expression was noted indicating that these antimicrobial genes, including the p38 MAPK pathway, have a significant role in *C. elegans* during *C. albicans* infection. Thus, PYR or TRI treatment enhanced the expression of antimicrobial genes that promote the elimination of *C. albicans* (Figure 7). 

## 3. Discussion

The development of new antifungal drugs remains a major challenge to overcoming the spread of drug-resistant clinically-relevant fungal pathogens, as strains are increasingly becoming less sensitive to conventional antifungal compounds [14,15].

In the present study, we selected two compounds, TRI and PYR, belonging to the triazine and pyridine chemical families, and showing antifungal activity against *C. albicans* and improved efficacy against fluconazole- or caspofungin-resistant clinical *Candida* strains.

Triazine is an important heterocyclic nucleus, which is a major component in some natural products such as reurhycin, toxoflavin, and fervenulin [16]. The chemistry of 1,2,4-triazines and their derivatives has attracted considerable attention owing to their broad spectrum of biological activity, including antifungal, anti-cancer, antiviral, cyclin-dependent kinase inhibitory, and anti-inflammatory activities [17,18,19,20,21]. Some 1,3,5-triazine derivatives, such as altretamine or almitrine have been approved by the FDA as antineoplastic agents or as respiratory stimulants, but none as antimicrobial agents. Moreover, very few reports have been published on the antimicrobial activity of such derivatives. In 1989, Reich et al. reported pyrido[3,4-e]-1,2,4-triazines and related heterocycles as having antifungal activity, with moderate activity against *C. albicans*, with MIC values ranging from 8 to 16 µg/mL for the lead compounds [22]. As shown in Figure 1, the reported compounds are bis-heterocycles and differ chemically from TRI.

Pyridin-2(1*H*)-compounds are naturally occurring products and display a variety of biological properties, including antimicrobial activity. Oxysporidinone was isolated after the fermentation of *Fusarium oxysporum* and has demonstrated antifungal activity against *Aspergillus niger*, *Botrytis cinerea*, *Alternaria alternata*, and *Venturia inequalis*, with MIC values of 10, 1, 50, and 10 µg/mL, respectively [23]. Its parent compound, funicolosin, is also a natural broad-spectrum antibiotic [24]. The related compound, ciclopirox, is a synthetic approved antifungal agent used as a topical treatment for superficial mycoses, particularly against *Tinea versicolor* [25]. As observed for TRI series, the compounds from the PYR series are chemically different from the reported oxysporidone, funicolosin, or ciclopirox parent molecules. Although these three reported antibiotics bear cyclic aliphatic moieties, PYR derivatives are composed of a pyridinone moiety decorated with two distinct aromatic rings.

Treatment with PYR and TRI resulted in a rapid reduction of *C. albicans* viability indicating that these compounds have fungicidal activity against *C. albicans.* Additionally, they reduced the viability of clinically isolated fluconazole- or caspofungin-resistant *Candida* strains. *C. albicans* cells in biofilms exhibit phenotypic traits, such as increased resistance to antifungal drugs and protection from host defenses that are dramatically different from their planktonic counterparts [5]. In the present study, we showed that challenge of *C. albicans* biofilms with either TRI or PYR contributed to a significant reduction in biofilm formation. Derivatives of PYR had low antifungal activity against *C. albicans* when compared to the original compound, while the derivative TRI1 did not have any antifungal activity at all.

The cell wall of *C. albicans* is the first point of contact between the fungus and the innate immune system [2]. The cell wall is composed of an outer layer enriched in mannan and mannosylated glycoproteins and an inner layer enriched in β-glucan and chitin [2,26]. We observed that PYR or TRI reduced the thickness of the mannan part of the *C. albicans* cell wall indicating that these two compounds can modulate the fungal cell wall and affect *C. albicans* virulence. However, no changes in chitin levels were observed in the fungal cells. These observations are consistent with our previous experimental studies, which showed that mannans were involved in *Candida* virulence and a deficiency in cell wall mannan contributed to a reduction in mouse mortality and intestinal inflammation induced by DSS [27].

In the *C. elegans* model, both TRI and PYR reduced the mortality of a high percentage of nematodes infected with *C. albicans* supporting the antifungal properties of these compounds against *C. albicans* infection [28]. *C. elegans* relies on its innate immune system to defend itself against fungal and bacterial pathogens by producing different antimicrobial proteins [29]. Caenacins and lysozyme, which are expressed in *C. elegans*, play a direct role against pathogens that infect worms via the intestinal lumen or cuticle [9,30,31]. Mallo et al. reported that overexpression of the lysozyme gene, lys-1, augmented the protection of *C. elegans* against *Serratia marcescens* infection [9]. In the current study, we showed that treatment with the two compounds enhanced the expression of antimicrobial genes (lys-1, lys-7, and cnc-4) in nematodes infected with *C. albicans*, indicating that PYR and TIR treatments not only had beneficial antifungal effects during infection but were also capable of improving the innate immune response of nematodes against *C. albicans* infection.

## 4. Materials and Methods

### 4.1. C. albicans Growth Conditions

*C. albicans* strain SC5314 used in this study was maintained at 4 °C in yeast peptone dextrose broth (YPD; 1% yeast extract, 2% dextrose, 2% peptone). To prepare the *C. albicans* suspension, fungal cells were cultured in Sabouraud dextrose broth (Sigma-Aldrich, St. Quentin Fallavier, France) for 24 h at 37 °C in a rotary shaker [32]. The fungal culture obtained was then centrifuged at 2500 rpm for 5 min and washed twice in PBS (phosphate-buffered saline). The clinical isolates were cultured in Sabouraud agar for 24–48 h. To identify these clinical isolates on the plates, the colonies were mixed with 1.5 μL of matrix solution (α-cyano-4-hydroxycinnamic acid; Bruker Daltonics, Bremen, Germany) dissolved in 50% acetonitrile, 47.5% water, and 2.5% trifluoroacetic acid, and analyzed by MALDI-TOF MS (Microflex-Bruker Daltonic, Bremen, Germany) [33,34].

### 4.2. Chemical Synthesis of PYR and TRI

**Figure 8:**
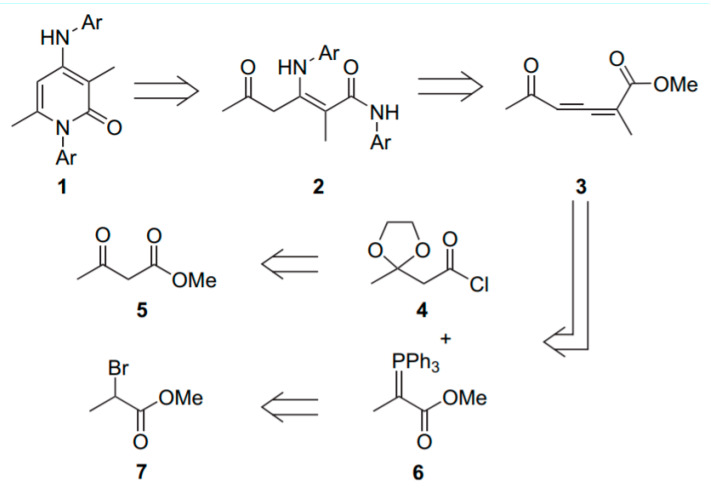
General pathway of PYR synthesis.

Compound PYR was synthesized as described previously by Boisse et al. [35]. The reaction was performed between an amine (4-chloroaniline for Cpd PYR) and an allenic precursor (Figure 8). This methodology led to new 1-aryl-3,6-dimethyl-4-aminoaryl-2-pyridones 1 in good yields. These syntheses can be performed in two distinct steps, allowing for the possibility of introducing different substituents in the positions 1 and 4. Using this strategy, all compounds from the PYR series (1) were obtained (Figure 8). All the procedures and chemical characterizations of PYR compounds are detailed in the publication described by Boisse et al. [35].

**Figure 9:**
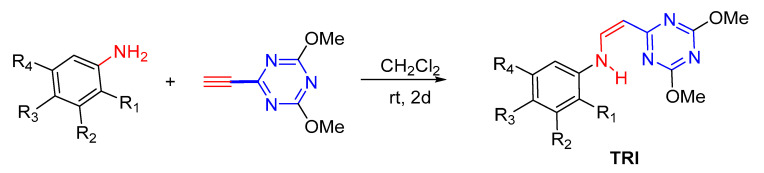
General pathway for the synthesis of TRI compounds.

The synthesis of TRI compounds has not yet been described in the literature. The full synthetic study will be reported in due course in a global article of organic chemistry. Briefly, TRI compounds were obtained by reaction of arylamine with 2-ethinyl-4,6-dimethoxy-1,3,5-triazine in dichloromethane under magnetic stirring at room temperature for 2 days (Figure 9).

### 4.3. Antifungal Compounds

PYR and TRI were designed, synthesized, and provided by JUNIA (a graduate school of general engineering-Hautes Etudes d’Ingénieur, Lille, France). Several small aliquots were prepared for each compound and stored in the freezer at −20 °C. These fresh aliquots were adjusted to the appropriate dilutions in PBS for each experiment. PYR and TRI were used at their minimum inhibitory concentrations (MICs; 12.5 µg/mL and 50 µg/mL, respectively), diluted in PBS during the various in vitro and in vivo experiments. Commercially available caspofungin (Merck, Semoy, France) and fluconazole (Fresenius, Sèvres, France) were used as positive controls. The negative control was PBS.

### 4.4. Fungal Viability Assays

MICs were measured using the broth microdilution method and Clinical Laboratory Standards Institute (CLSI) procedures [36]. The screening was carried out by CO-ADD (Community for Antimicrobial Drug Discovery) and the University of Queensland (Santa Lucia, Australia). After the culture of *C. albicans* ATCC 90028 for 3 days on YPD agar at 30 °C, a *C. albicans* suspension of 1 × 10^6^ to 5 × 10^6^ CFU/mL was prepared [12]. This suspension was next diluted and added to each well of the PYR- or TRI-compound-containing plates giving a final cell density of fungi of 2.5 × 10^3^ CFU/mL in a total volume of 50 μL. All plates were covered and incubated at 35 °C for 36 h without shaking. After the addition of resazurin (0.001% final concentration) and incubation at 35 °C for 2 h, the growth inhibition of *C. albicans* ATCC 90028 was assessed by measuring absorbance at 630 nm. The absorbance was assessed using a Biotek Multiflo Synergy HTX plate-reader. The percentage growth inhibition was assessed for each well, using the negative control (media only) and positive control (fungi without inhibitor) on the same plate. The MIC was determined as the lowest concentration at which fungal growth was completely inhibited (Table 1) [12], defined by inhibition of ≥80% for *C. albicans* (total inhibition in the case of PYR at 12.5 µg/mL and TRI at 50 µg/mL). These two compounds showed no cytotoxicity at fungicidal concentrations on different human lung, breast, renal, prostate, or colon cell lines [37]. A bioluminescent *C. albicans* strain was used for the viability assays [38]. This bioluminescent strain was suspended in PBS at a volume of 10^6^ cells/well (96-well black plates, Chimney well). PYR, TRI, caspofungin, or fluconazole were then added at their final MIC (Table 1). The coelenterazine substrate was then added to the wells at a concentration of 2 μM [12]. Bioluminescence kinetics were then measured (at 0, 30, 60, and 120 min) and analyzed with a FLUOstar Omega Fluorometer (BMG Labtech, Champigny sur Marne, France). The positive control consisted of *C. albicans* challenged with PBS only. The viability of *C. albicans* was also evaluated by culture assay in the presence of PYR, TRI, fluconazole, and caspofungin. *C. albicans* strain SC5314 was suspended in PBS at a concentration of 10^5^ *C. albicans* cells and antifungal compounds were then added. PYR, TRI, fluconazole, and caspofungin were used at their MIC (12.5 µg/mL, 50 µg/mL, 0.5 µg/mL, and 0.03 µg/mL, respectively). Serial dilutions ranging from 10^−1^ to 10^−4^ were performed on the samples suspended in PBS and 100 μL of each dilution was spread onto Sabouraud agar at 37 °C.

### 4.5. Effect of PYR and TRI on C. albicans Biofilm Formation

*C. albicans* at a concentration 10^7^ colony-forming units (cfu) in 200 µL of RPMI was added to each well of a 96-well polystyrene plate (Greiner Bio-one) and incubated for 24 h. PYR and TRI were then added to the plate at their 1× MIC (12.5 µg/mL and 50 µg/mL, respectively) for 24 h. Caspofungin or fluconazole were also used at their MICs as positive controls. After three washes with PBS to remove non-adherent cells, 110 μL of crystal violet (0.4%; Fluka) was added to each well. After three washes with PBS, crystal violet staining was decolorized by adding 200 µL of ethanol to the plate. The absorbance of the decolorization solution that reflected the numbers of viable cells was measured at 550 nm using a spectrophotometer (FLUOstar; BMG Labtech, Champigny sur Marne, France). The results are presented as the mean of six replicates from two independent experiments.

### 4.6. C. elegans Survival Assay and RT-PCR Quantification of Antimicrobial Genes of C. elegans

*C. albicans* SC5314 strain was inoculated in 2 mL of Sabouraud broth and incubated at 36 °C for 24 h. *Candida* lawns were prepared by spreading 10 µL of the *C. albicans* culture onto plates containing solid BHI medium with amikacin (45 µg/mL). These plates were then incubated at 36 °C for 24 h. N2 wild-type *C. elegans* was grown on nematode growth medium seeded with *E. coli* OP50 as a food source at room temperature for 72 h, until they reached the L4 stage. Approximately 100 worms were selected and washed with M9 buffer containing 90 µg/mL amikacin to eliminate *E. coli*. These worms were then picked onto the *Candida* lawn and allowed to feed for 6 h. The nematodes were washed three times with M9 buffer to remove *C. albicans* cells from their cuticles. Approximately 70–80 nematodes were then picked to wells in a 6-well microtiter dish that contained 2 mL of liquid 80% M9 buffer, 20% BHI, 10 µg/mL cholesterol in ethanol, and 90 µg/mL amikacin. TRI and PYR at their MICs (12.5 µg/mL and 50 µg/mL, respectively) were added to each well. The plates were incubated at room temperature for 12 h. The worms were examined for survival daily for 4 days. Nematodes were considered dead when they did not respond to being touched by a platinum-wire pick.

For the RT-PCR assay, total RNA was isolated from N2 after 12 h of antifungal treatment using a NucleoSpin RNA^®^ kit (Macherey-Nagel, Hoerdt, France). Nematode RNA was quantified by spectrophotometry (Nanodrop; Nyxor Biotech, Paris, France). cDNA synthesis was carried out using a High Capacity DNA Reverse Transcription (RT) kit, with Master Mix (Applied Biosystems, CA, USA). To amplify the cDNA, Fast SYBR green (Applied Biosystems) was employed in a one-step system (Applied Biosystems) [39]. SYBR green dye intensity was assessed using one-step software. All results were normalized to the reference gene, *act-2*.

### 4.7. Statistical Analysis

The Mann–Whitney U test was used to analyze the differences between the groups and the results were considered to be statistically significant when the *p* value was as follows: *p* < 0.05; *p* < 0.01; *p* < 0.001. All statistical analyses were carried out using GraphPad Prism version 6 (GraphPad, La Jolla, CA, USA).

## 5. Conclusions

In conclusion, two series of triazine (TRI) and pyridinone (PYR) derivatives were selected for their antifungal potential. These series share some similarities such as a 6-membered *N*-heterocycle, a free -NH- link and a para-substituted aromatic moiety. Whereas in series TRI, the *p*-methoxyaniline was the most effective ring for antifungal activity, it appeared that in series PYR, *p*-chloroaniline was the best choice. TRI and PYR were found to have fungicidal activity against *C. albicans* which was more pronounced than their analogues, and both reduced the viability of clinically isolated fluconazole- or caspofungin-resistant *Candida* strains. PYR and TRI decreased *C. albicans* biofilm formation and decreased the thickness of the mannan part of the cell wall. In a *C. elegans* nematode infection model, PYR and TRI decreased the mortality of nematodes infected with *C. albicans* and enhanced the expression of antimicrobial genes that promote the elimination of *C. albicans.* Overall, this study provides evidence that PYR and TRI are able to reduce the viability of drug-resistant *C. albicans* clinical isolates and offer promising therapeutic applications against biofilm-associated *C. albicans* infections. The identification of these two compounds PYR and TRI will enable us to widen our study to in silico approaches and to predict the possible binding sites of these two compounds in due course.

## Figures and Tables

**Table 1:**
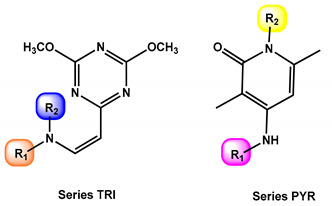
Chemical modifications of TRI and PYR and their impact on antifungal activity.

Entry	CPD	R_1_	R_2_	%Inhibition at 32 µg/mL^a^	%Inhibition at 0.8 mmol/L (µg/mL) ^b^
1	TRI	4-CH_3_O-C_6_H_4_-	H	32.1	98 (230)
2	TRI1	C_6_H_5_-	H	3.9	72 (206)
3	TRI2	2,5-(CH_3_O)_2_-C_6_H_4_-	H	1.7	77 (255)
4	TRI3	2,4-(CH_3_O)_2_-C_6_H_4_-	H	0.5	ND
5	TRI4	3,4,5-(CH_3_O)_3_-C_6_H_4_-	H	1.9	ND
6	TRI5	3-(OH),4-(CH_3_O)-C_6_H_4_-	H	0.8	ND
7	TRI6	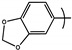	H	1.4	ND
8	TRI7	3,5-(CH_3_O)_2_-C_6_H_4_-	H	3.9	ND
9	TRI8	3,5-(Cl)_2_-C_6_H_4_-	H	0.5	ND
10	TRI9	CH_3_-CH_2_-	CH_3_-CH_2_-	3.8	84 (190)
11	PYR	4-Cl-C_6_H_4_-	4-Cl-C_6_H_4_-	14.0	98 (287)
12	PYR1	4-CH_3_-C_6_H_4_-	4-CH_3_-C_6_H_4_-	8.1	88 (255)
13	PYR2	C_6_H_5_-	C_6_H_5_-	3.3	ND
14	PYR3	4-CH_3_O-C_6_H_4_-	4-CH_3_O-C_6_H_4_-	4.2	ND
15	PYR4	3,4-(CH_3_O)_2_-C_6_H_4_-	3,4-(CH_3_O)_2_-C_6_H_4_-	2.5	ND
16	PYR5	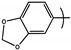	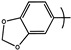	3.4	ND
17	PYR6	3,4-(CH_3_O)_2_-C_6_H_4_-	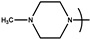	3.0	ND
18	PYR7	3,4-(CH_3_O)_2_-C_6_H_4_-	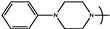	3.6	ND

Biological activity was evaluated at a concentration of ^a^ 32 µg/mL or ^b^ 0.8 mmol/L corresponding to 230, 206, 255, 190, 287, and 255 µg/mL for TRI, TRI1, TR2, TRI9, PYR, and PYR1, respectively, on *C. albicans* strain ATCC90028 grown for 3 days on yeast extract–peptone dextrose (YPD) agar at 30 °C. A yeast suspension of 1 × 10^6^ –5 × 10^6^ cfu/mL (determined by OD_550_) was prepared from five colonies. The suspension was diluted and added to each well of the compound-containing plates to give a final fungal cell concentration of 2.5 × 10^3^ cfu/mL and a total volume of 50 µL. All plates were covered and incubated at 35 °C for 24 h without shaking. Inhibition of *C. albicans* growth was determined by measuring the absorbance at 530 nm (OD_530_). Fluconazole was used as a positive control. CPD: compound. ND: not determined.

**Table 2:** Antifungal activity of PYR and TRI versus caspofungin and fluconazole.

Strain	Description	MIC Caspofungin µg/mL	MIC Fluconazole µg/mL	MIC PYR µg/mL	MIC TRI µg/mL	Strain Ref.
*C. albicans *SC5314	Wild-type	0.03	0.5	12.5	50	[13]
Bioluminescent *C. albicans *	*C. albicans * strain CAI4 (ura3::imm434/ura3::imm434)	0.03	0.5	12.5	50	[12]
*C. albicans * 17292c3367	Venous catheter, caspofungin-resistant	8	0.5	12.5	50	This study
*C. albicans * 15343c3523	Blood, caspofungin-resistant	2	0.5	12.5	50	This study
*C. albicans * 15351c6859	Venous catheter, caspofungin-resistant	4	1	12.5	50	This study
*C. albicans * 14402c5521	Abdominal lesions, fluconazole-resistant	0.125	256	12.5	50	This study

**Table 3:** Effect of PYR and TRI on the viability of drug-resistant *C. albicans* clinical isolates.

Strain	Viability of *C. albicans* at 2× MIC (µg/mL)		Viability of *C. albicans* at 4x MIC (µg/mL)	
	TRI vs. Caspo (%)	TRI vs. Fluco (%)	PYR vs. Caspo (%)	PYR vs. Fluco (%)
Caspofungin-resistant *C. albicans* 15343c3523	14.81 ± 7.8 vs. 83.56 ± 32.05 *		24.96 ± 2.23 vs. 33.47 ± 18.6 *	
Caspofungin-resistant *C. albicans* 15351c6859	13.49 ± 17.04 vs. 194.03 ± 8.014 *		22.08 ± 13.95 vs. 231.89 ± 50.58 *	
Caspofungin-resistant *C. albicans* 17292c3367	29.54 ± 17.8 vs. 130.76 ± 6.101 *		10.79 ± 8.04 vs. 125.11 ± 62.04 *	
Fluconazole-resistant *C. albicans* 14402c5521		11.30 ± 13.2 vs. 33.68 ± 26.2 *		10.95 ± 9.49 vs. 29.12 ± 20.03 *

* *p* < 0.05.

## Data Availability

The data presented in this study are available in the article.
